# Noninvasive imaging of vascular permeability to predict the risk of rupture in abdominal aortic aneurysms using an albumin-binding probe

**DOI:** 10.1038/s41598-020-59842-2

**Published:** 2020-02-24

**Authors:** Lisa C. Adams, Julia Brangsch, Carolin Reimann, Jan O. Kaufmann, Kristin Nowak, Rebecca Buchholz, Uwe Karst, Rene M. Botnar, Bernd Hamm, Marcus R. Makowski

**Affiliations:** 1 Charité – Universitätsmedizin Berlin, corporate member of Freie Universität Berlin, Humboldt-Universität zu Berlin, and Berlin Institute of Health, Charitéplatz 1, 10117 Berlin, Germany; 2 Department of Veterinary Medicine, Institute of Animal Welfare, Animal Behavior and Laboratory Animal Science, Freie Universität Berlin, Königsweg 67, Building 21, 14163 Berlin, Germany; 3 Division 1.5 Protein Analysis, Federal Institute for Materials Research and Testing (BAM), Richard-Willstätter-Str. 11, 12489 Berlin, Germany; 4 Department of Chemistry, Humboldt-Universität zu Berlin, Brook-Taylor-Str. 2, 12489 Berlin, Germany; 5 Institute of Inorganic and Analytical Chemistry, Westfälische Wilhelms-Universität Münster, Corrensstr. 30, 48149 Münster, Germany; 6 King’s College London, School of Biomedical Engineering and Imaging Sciences, United Kingdom, St Thomas’ Hospital Westminster Bridge Road, London, SE1 7EH United Kingdom; 7 BHF Centre of Excellence, King’s College London, United Kingdom, Denmark Hill Campus, 125 Coldharbour Lane, London, SE5 9NU United Kingdom

**Keywords:** Predictive markers, Experimental models of disease

## Abstract

Abdominal aortic aneurysm (AAA) remains a fatal disease. Its development encompasses a complex interplay between hemodynamic stimuli on and changes in the arterial wall. Currently available biomarkers fail to predict the risk of AAA rupture independent of aneurysm size. Therefore, novel biomarkers for AAA characterization are needed. In this study, we used a mouse model of AAA to investigate the potential of magnetic resonance imaging (MRI) with an albumin-binding probe to assess changes in vascular permeability at different stages of aneurysm growth. Two imaging studies were performed: a longitudinal study with follow-up and death as endpoint to predict rupture risk and a week-by-week study to characterize AAA development. AAAs, which eventually ruptured, demonstrated a significantly higher *in vivo* MR signal enhancement from the albumin-binding probe (p = 0.047) and a smaller nonenhancing thrombus area compared to intact AAAs (p = 0.001). The ratio of albumin-binding-probe enhancement of the aneurysm wall to size of nonenhancing-thrombus-area predicted AAA rupture with high sensitivity/specificity (100%/86%). More advanced aneurysms with higher vascular permeability demonstrated an increased uptake of the albumin-binding-probe. These results indicate that MRI with an albumin-binding probe may enable noninvasive assessment of vascular permeability in murine AAAs and prediction of rupture risk.

## Introduction

Abdominal aortic aneurysm (AAA) poses an increasing burden on healthcare systems and is among the challenges currently facing society, with prevalence ranging from 5% to 10% in Western populations^1^. AAA development is a multifactorial, mainly degenerative process encompassing a complex interplay between hemodynamic stimuli on and changes in the arterial wall, including endothelial dysfunction^2–7^. Rupture of AAA is a life-threatening complication and surgical emergency with high mortality^1^. While larger (≥55 mm), symptomatic, or fast-growing (>10 mm/year) AAAs are recommended to undergo endovascular or operative repair as the only available treatment options, patients with smaller AAAs, who are considered to have a lower risk of rupture, are often monitored with non-invasive imaging^8^. By contrast, the management of medium-sized AAAs remains controversial^9^. Apart from diameter and growth rate, there is currently no recognized biomarker for evaluating AAA characteristics and rupture risk^1^. The main limitation of aneurysm diameter as the primary criterion for intervention is that it fails to account for smaller aneurysms with high rupture risk and also for large unruptured AAAs^10^. This is further complicated by the scarcity of longitudinal data on ruptured AAAs, as most patients are followed up until meeting criteria for intervention, while prior AAA existence is often unknown in patients presenting with rupture.

Therefore, novel biomarkers for the characterization of AAA development and identification of individuals at risk for rupture are needed^11^. Noninvasive magnetic resonance imaging (MRI) with its high spatial resolution for evaluation of the aortic wall and aneurysm components is a promising tool in this context^12^. When combined with target-binding probes, MRI can additionally detect and characterize pathological processes *in vivo*. Gadofosveset trisodium is a clinically approved gadolinium-containing MRI probe that reversibly binds to albumin in the arterial and venous system. In case of increased vascular permeability, it may enter the vessel wall and interstitium through leaky neovessels or damaged endothelium, as previously demonstrated in an animal model of atherosclerosis^13,14^.

Based on a mouse model of AAA, this study investigates, firstly, if *in vivo* MRI with gadofosveset could allow assessment of vascular permeability at different stages of AAA and, secondly, if it might allow prediction of AAA rupture.

## Results

**Figure 1:**
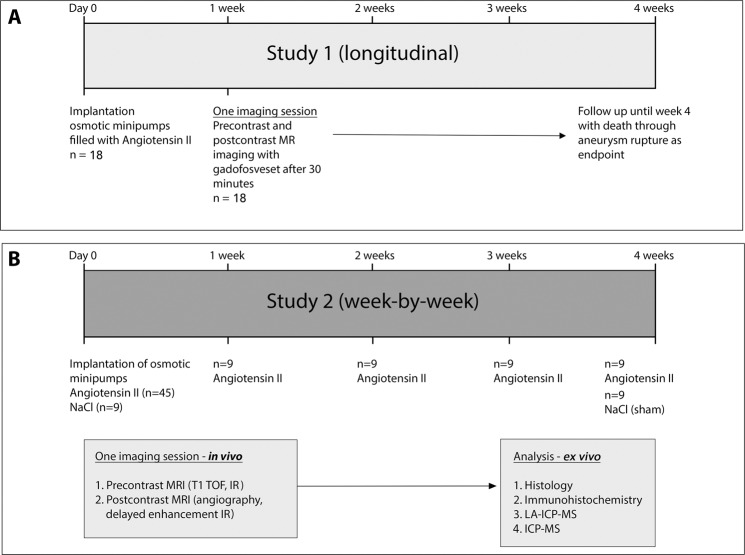
Experimental setup. (**A**) For study 1 (longitudinal), a single MRI session was performed after one week (n = 18) with injection of a clinical dose of the albumin-binding probe gadofosveset. After imaging, animals were followed up for up to four weeks with death by aneurysm rupture as endpoint. The aim was to evaluate the potential of gadofosveset to predict aneurysm rupture. (**B**) In study 2 (week-by-week study), imaging after administration of gadofosveset (0.03 mmol/kg) was performed after 1, 2, 3, and 4 weeks (n = 9 per group) of angiotensin II infusion. *Ex vivo* analysis (histology, fluorescence immunohistochemistry, ICP-MS and LA-ICP-MS) was performed after the final scan of each week (n = 9 per week) to correlate *in vivo* with *ex vivo* results. As control, nine sham-treated ApoE^−/−^ mice were implanted with osmotic minipumps primed with sodium chloride.

**Figure 2:**
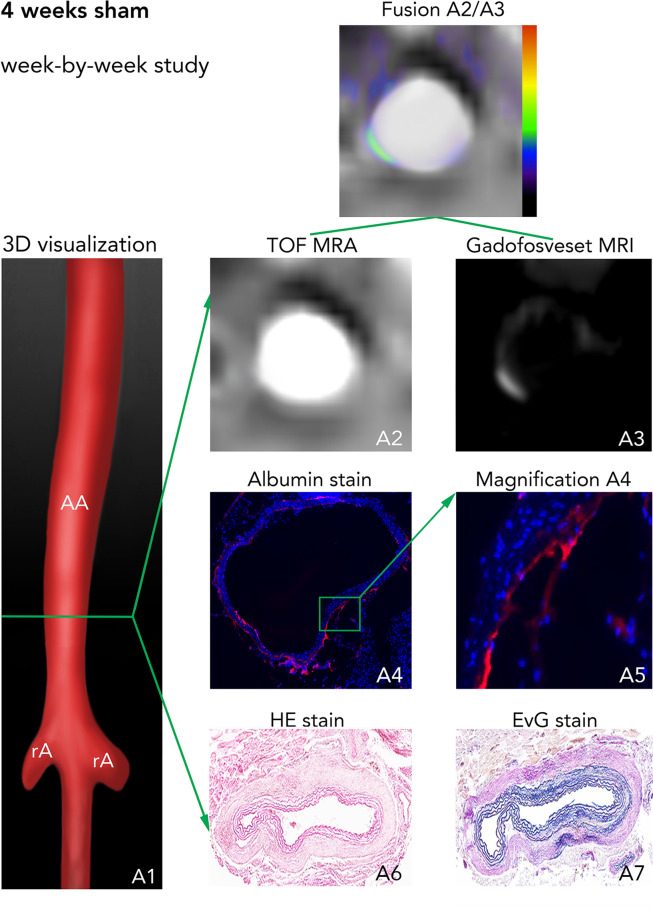
*In vivo* MRI with an albumin-binding probe and *ex vivo* assessment in sham animals. (A1) 3D visualization showing the suprarenal abdominal aorta (AA) with the renal arteries (rA) of a male ApoE^−/−^ mouse after 4 weeks of saline infusion. The green line indicates the orientation of subsequently acquired transverse MRI sequences and the corresponding histological sections. (A2) Time-of-flight angiography of the AA. (A3) Delayed-enhancement imaging after administration of the albumin-binding probe gadofosveset. Fusion A2/A3 shows a fusion of images A2 and A3 for better visualization of *in vivo* results. A4-A7 are images of *ex vivo* histology. A4 and its corresponding magnification A5 show an albumin-specific staining with only minor immunopositive fluorescent areas of albumin. A6 corresponds to a Hematoxylin and Eosin (H&E) staining of the aortic tissue and A7 shows an Elastica van Gieson (EvG) staining of elastic fibers. In summary, we see, that no pathological changes of the aortic wall can be observed *in vivo* based on delayed-enhancement imaging with the albumin-binding probe gadofosveset (A3, A2/A3) or on corresponding *ex vivo* histology (A4-7). *Abbreviations:* H&E: Hematoxylin and Eosin staining; EvG: Elastica van Gieson staining of elastic fibers; TOF-MRA: time-of-flight magnetic resonance angiography; AA: suprarenal abdominal aorta; RA: right renal artery; gadofosveset: albumin-binding probe. +: vascular lumen in arterial TOF.

Refer to Fig. 1 for details of the study design. Mice were 12 ± 2 weeks old and drug-aïve with a weight between 27 g and 32 g (see Table 1). Sham-treated mice with a continuous saline infusion for 4 weeks served as controls and did not show any signs of AAA development (see Fig. 2).

**Table 1:** Baseline animal characteristics in each group.

Group	Age (weeks)	Weight (g)
Sham-treated	12 ± 2	28 ± 1
1 week	12 ± 2	29 ± 3
2 weeks	12 ± 2	27 ± 2
3 weeks	12 ± 2	30 ± 1
4 weeks	12 ± 1	29 ± 3
Longitudinal	12 ± 1	27 ± 2

### Longitudinal study for prediction of AAA rupture

**Figure 3:**
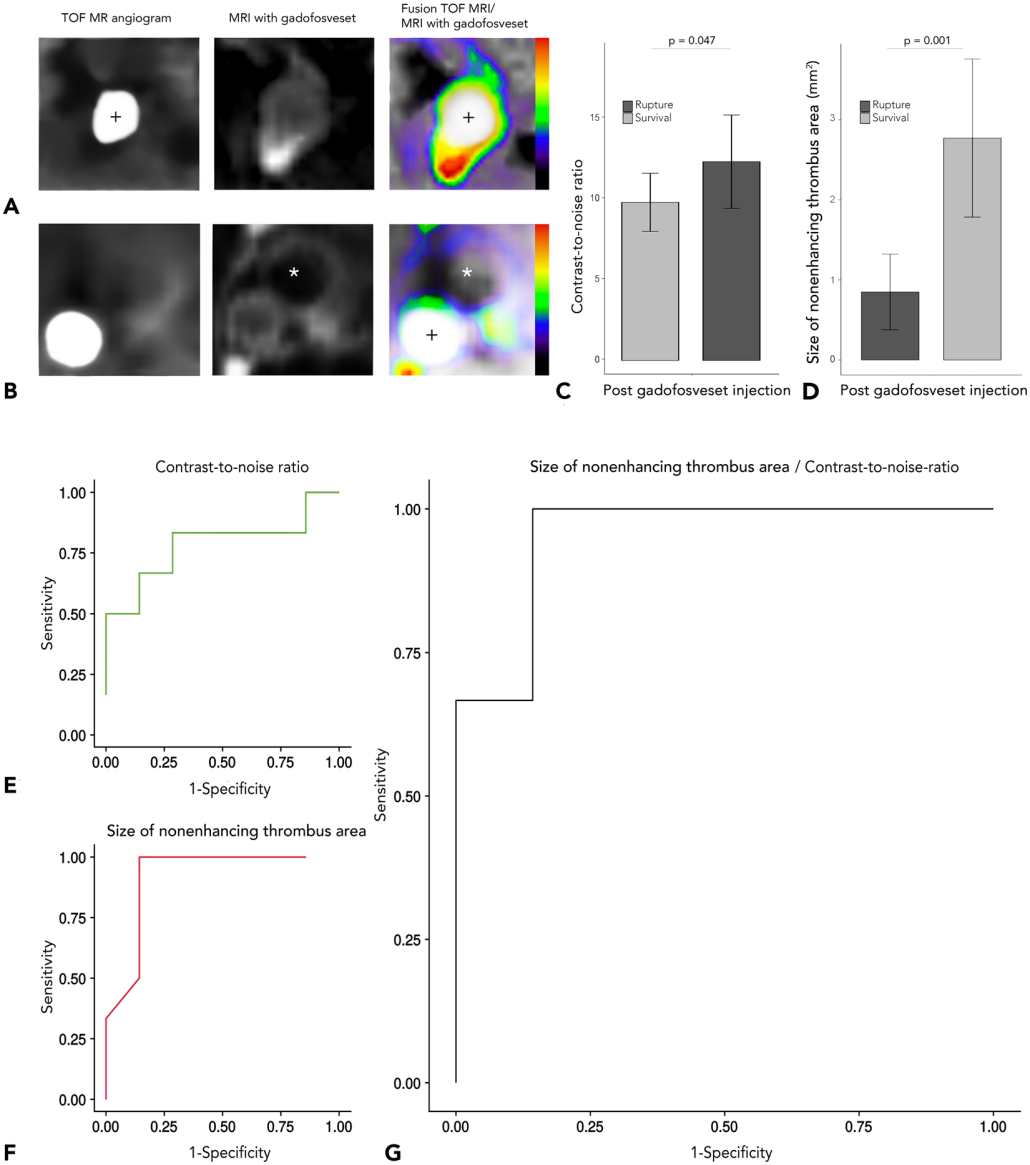
Assessment of aneurysm rupture in animals with stable aortic aneurysm and in animals with fatal aortic rupture (longitudinal study). (**A**,**B**) Cross-sectional time-of-flight (TOF) angiographies (on the left), delayed-enhancement sequences after administration of the albumin-binding probe gadofosveset (centre) and delayed-enhanced images fused with magnetic resonance TOF angiography (right). These images show the suprarenal abdominal aorta of an ApoE^−/−^ mouse with AAA rupture (**A**) compared to a surviving ApoE^−/−^ mouse. (**B**) In the fusion images on the right, blue signal indicates little enhancement, whereas red signal indicates increased enhancement following administration of an albumin-binding probe. We see, that ApoE^−/−^ mice with AAA rupture show significantly increased signal enhancement, as measured by contrast-to-noise ratio (p = 0.047, see (**C**) and a smaller nonenhancing thrombus area (p = 0.001, see (**D**)). (**E**-**G**) show ROC curves for contrast-to-noise ratio, size of nonenhancing thrombus and a combination of both. (**E**) We see, that use of the signal from the albumin-binding probe (contrast-to-noise ratio) predicted AAA rupture risk with 80% sensitivity and 70% specificity. (**F**) Size of the nonenhancing thrombus area predicted AAA rupture with 100% sensitivity and 78% specificity. (**G**) The combined used of both parameters had the highest accuracy for predicting aneurysm rupture with 100% sensitivity and 86% specificity compared with each parameter alone. *Abbreviations:* Gadofosveset: albumin-binding probe. *Nonenhancing intraluminal thrombus area; ROC: Receiver operating characteristic; TOF-MRA: time-of-flight magnetic resonance angiography; +: vascular lumen in arterial TOF.

To evaluate the potential of delayed-enhancement MRI using gadofosveset as a prognostic tool for aneurysm rupture, we performed a longitudinal study of ApoE^−/−^ mice with gadofosveset imaging after 1 week of AngII infusion and with fatal aneurysm rupture as the endpoint (study 1, n = 18). After one week, we observed that, while there was no significant difference in aneurysm size (p = 0.061), increased enhancement (CNR) of the aneurysm wall in combination with the absence of nonenhancing intraluminal thrombus was associated with an increased rupture risk (see Fig. 3). Thus, the risk of rupture could be predicted most accurately by assessing gadofosveset enhancement (CNR) in conjunction with the area of nonenhancing organized thrombus (p = 0.009). While surviving animals showed CNR values of 9.74 ± 1.94 and a nonenhancing thrombus area of 2.77 mm^2^ ± 1.07 mm^2^, animals with rupturing aneurysms had CNR values of 12.25 ± 2.74 and a nonenhancing thrombus area of 0.80 mm^2^ ± 0.51 mm^2^ (difference in CNR: p = 0.047, difference in nonenhancing thrombus area: p = 0.001). The ratio of CNR to the size of the nonenhancing thrombus area predicted fatal AAA rupture with 100% sensitivity and 86% specificity (see Fig. 3G).

### Abdominal aortic aneurysm progression and gadofosveset uptake

In the week-by-week study, ApoE^−/−^ mice with AAAs showed an average increase of 154% ± 12% in aortic diameter after 1 week, of 168% ± 15% after 2 weeks, and of 243% ± 19% after 4 weeks (Supplemental Fig. 1). After implantation of osmotic minipumps, approximately 25% of ApoE^−/−^ mice died before imaging but none of the sham-treated ApoE^−/−^ mice.

**Figure 4:**
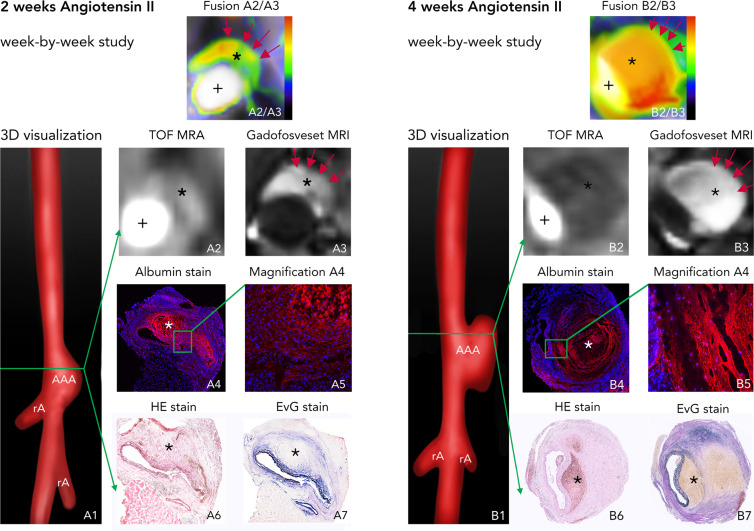
*In vivo* molecular MRI with an albumin-binding probe during the development of aortic aneurysms (week-by-week study). (A1) 3D visualizations showing the suprarenal abdominal aorta (AA) with the renal arteries (rA) of a male ApoE^−/−^ mouse after 2 weeks of Angiotensin II infusion. The green line indicates the orientation of subsequently acquired transverse MRI sequences and the corresponding histological sections. (A2) Time-of-flight angiography of the AA showing the vessel lumen. (A3) Delayed-enhancement imaging after administration of the albumin-binding probe gadofosveset shows intermediate signal enhancement after 2 weeks of AngII infusion, indicated by the red arrows and corresponding to albumin accumulation in the arterial wall and the thrombus area. Fusion A2/A3 shows a fusion of images A2 and A3 for better visualization of *in vivo* results. Blue signal indicates lower enhancement, whereas green signal shows intermediate enhancement and red signal indicates increased enhancement. A4–A7 are images of *ex vivo* histology. A4 and its corresponding magnification A5 show an albumin-specific staining with confluent immunopositive fluorescent areas of albumin, confirming the extraluminal accumulation of albumin. A6 corresponds to a Hematoxylin and Eosin (H&E) staining of the aortic tissue and A7 shows an Elastica van Gieson (EvG) staining of elastic fibers. In summary, we see, that there is pathological vascular permeability in the abdominal aortic aneurysm after 2 weeks of Angiotensin II infusion with intermediate signal enhancement from the albumin-binding probe, corresponding to confluent extraluminal accumulation of Albumin. (B1) 3D visualizations showing the suprarenal abdominal aorta (AA) with the renal arteries (rA) of a male ApoE^−/−^ mouse after 4 weeks of Angiotensin II infusion. The green line indicates the orientation of subsequently acquired transverse MRI sequences and the corresponding histological sections. (B2) Time-of-flight angiography of the AA showing the vessel lumen. (B3) Delayed-enhancement imaging after administration of the albumin-binding probe gadofosveset shows a strong signal enhancement after 4 weeks of AngII infusion, indicated by the red arrows and corresponding to albumin accumulation in the arterial wall and the thrombus area. Fusion B2/B3 shows a fusion of images B2 and B3 for better visualization of *in vivo* results. Blue signal indicates lower enhancement, whereas green signal shows intermediate enhancement and red signal indicates increased enhancement. B4-B7 are images of *ex vivo* histology. B4 and its corresponding magnification B5 show an albumin-specific staining with large immunopositive fluorescent areas of albumin, confirming an increasing extraluminal accumulation of albumin. B6 corresponds to a Hematoxylin and Eosin (H&E) staining of the aortic tissue and B7 shows an Elastica van Gieson (EvG) staining of elastic fibers. In summary, we see, that there is a further increase in vascular permeability in the abdominal aortic aneurysm after 4 weeks of Angiotensin II infusion with strong enhancement from the albumin-binding probe, corresponding to extensive extraluminal accumulation of Albumin. *Abbreviations:* H&E: Hematoxylin and Eosin staining; EvG: Elastica van Gieson staining of elastic fibers; TOF-MRA: time-of-flight magnetic resonance angiography; gadofosveset: albumin-binding probe; AA: suprarenal abdominal aorta; RA: right renal artery; gadofosveset: albumin-binding probe; +: vascular lumen in arterial TOF; *: thrombus area.

**Figure 5:**
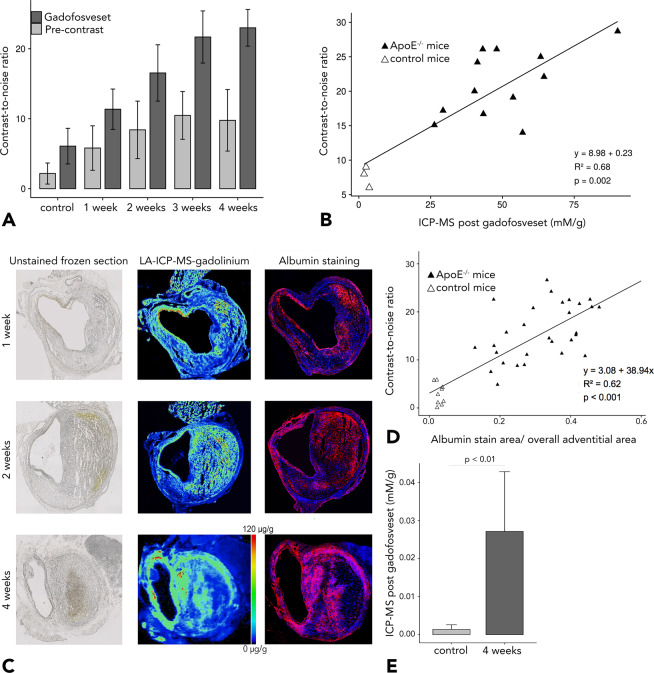
*Ex vivo* quantification of *in vivo* MRI results. (**A**) Contrast-to-noise ratios measured in control animals and in ApoE^−/−^ mice after 1, 2, 3 and 4 weeks of Angiotensin II infusion. Postcontrast (gadofosveset) contrast-to-noise ratios show increased signal enhancement with progression of abdominal aortic aneurysm (AAA) in ApoE^−/−^ mice. (**B**) Shows a significant positive correlation between gadofosveset-enhanced contrast-to-noise ratios and gadofosveset-based gadolinium concentrations as determined by inductively coupled plasma mass spectrometry (ICP-MS) (Pearson’s correlation coefficient of R^2^ = 0.68). (**C**) Demonstrates the unstained frozen section (left), the spatial distribution of gadolinium (from the albumin-specific gadolinium-based probe) in aortic aneurysms as assessed by LA-ICP-MS (middle) and the co-localization of albumin-positive areas in the fluorescence albumin stain with gadolinium distribution (left). (**D**) *Ex vivo* relative fluorescence albumin-stained areas also demontrate significant correlation with *in vivo* measured contrast-to-noise ratios (R^2^ = 0.62). (**E**) Compared to sham-treated animals, Angiotensin II-infused ApoE^−/−^ mice at 4 weeks show significantly higher gadolinium content as determined by ICP-MS. n = 9 per group for the MRI experiments. n = 3 per group for ICP-MS.

On postcontrast, delayed-enhancement MRI, greater enhancement was observed in ApoE^−/−^ mice with AAAs compared to sham-treated mice not developing AAA. CNR values were higher for ApoE^−/−^ mice with 4 weeks of AngII infusion compared to mice with 2 weeks of AngII infusion (Fig. 4). There was an increase in the CNR of the aneurysm region with progression of AAAs in ApoE^−/−^ mice (see Fig. 5A). Histology confirmed *in vivo* findings, revealing dilatation of the aortic lumen and formation of intraluminal thrombus (see Fig. 4*A4-7 and B4-7*). Remodelling of the rupture site and adjacent aneurysmal wall was most pronounced in late-stage aneurysms (after 4 weeks of AngII infusion). By contrast, signal enhancement was decreased in aneurysm-free sham-treated ApoE^−/−^ mice. There was a significant correlation between CNR in the aneurysmal area and gadolinium concentration, as determined by ICP-MS (Fig. 5B).

### Albumin measurement

Only minor immunopositive fluorescent areas of albumin were found in the aorta of sham-treated ApoE^−/−^ mice, especially in the adventitia. By contrast, in AngII-infused ApoE^−/−^ mice, large immunopositive fluorescent areas were observed in the aneurysm wall with higher intensity and larger fluorescent areas (Fig. 5C). The correlation of fluorescent-albumin-containing areas with contrast-to-noise ratios (p < 0.001) is illustrated in Fig. 5D. Albumin areas were significantly larger after 2 and 4 weeks (24% ± 4% and 49% ± 7%, respectively) compared with sham-treated ApoE^−/−^ mice (9% ± 5%, p < 0.001).

### Gadolinium concentration as determined by inductively coupled mass spectrometry

Absolute gadolinium concentrations in AAAs were measured by ICP-MS in 18 mice (n = 3 per time point (week-by-week study) and n = 3 sham-treated animals). There was a strong correlation of *in vivo* CNRs with gadolinium concentrations measured *ex vivo* as a surrogate for aneurysmal albumin (Fig. 5B).

### Spatial localization with laser ablation inductively coupled plasma mass spectrometry

LA-ICP-MS was used for direct visualization of spatial gadofosveset-associated gadolinium distribution in histological samples. AAA sections of ApoE^−/−^ mice with 1, 2, and 4 weeks of AngII infusion and with 4 weeks of AngII infusion showed strong co-localization of targeted gadolinium with immunopositive fluorescent areas of albumin (Fig. 5). Consistent with the findings for immunofluorescence albumin staining, an increase in gadolinium concentration was observed in ApoE^−/−^ mice with 4 weeks of AngII infusion versus 1 week of AngII infusion. Similarly, there was an increase of Evans Blue Dye in the aortic tissue of ApoE^−/−^ mice with 4 weeks of angiotensin II infusion compared to control animals (*also refer to* Supplemental Fig. 2A). We could demonstrate, that the uptake of gadofosveset correlated with the amount of Evans Blue dye in the aortic tissue (Supplemental Fig. 2B). Therefore, our results suggest that AAA are associated with vascular permeability and subsequent extraluminal albumin accumulation. This may be used as a biomarker for assessing the rupture risk in murine AAA.

## Discussion

In this study, we report a novel approach for the assessment of vascular permeability and the prediction of aneurysm rupture in a murine model of AAA, using an albumin-binding MR probe. AAAs, which ruptured during this study, had significantly higher *in vivo* MR signal enhancement from the albumin-binding probe compared to unruptured AAAs. We calculated a ratio of albumin-binding probe enhancement (CNR) of the aneurysm wall to the size of nonenhancing thrombus area, which allowed us to predict AAA rupture with high sensitivity and specificity.

Previous *ex vivo* studies demonstrated more marked vascular permeability in patients with AAAs and found an association with a higher rupture risk^4,5^.

Following intravenous administration, gadofosveset binds to albumin in the vascular system, resulting in a 5- to 10-fold increase in T1 relaxivity compared to standard gadolinium-based contrast agents. If the endothelial barrier between blood and the extravascular space is not intact, e.g., as a result of the loss of endothelial cells, albumin-bound gadofosveset leaks into the extravascular aneurysm wall. This process can be visualized with high spatial resolution using molecular MRI techniques.

AAAs are often associated with intraluminal thrombus (ILT)^15^. The impact of ILT on AAA rupture is still controversial and therefore currently unclear, with partly contradictory study results^16^. While a mechanical view considers the intraluminal thrombus as a protective layer, reducing mechanical stress in the aortic wall and maintaining its stability^17,18^, another concept focus on the intraluminal thrombus as a biologically active entity. According to this concept, the secreted molecules contribute to an inflammatory response and proteolytic injury of the arterial wall, culminating in aortic thinning and expansion^19^. The concept of these two approaches might be extended by assuming the presence of intraluminal thrombi with different properties. *Zhu et al*. found brighter, presumably fresher and less organized degraded thrombus to be associated with more rapid aneurysm growth, agreeing with another previous study^12,20^. The results of our study might also be put into this context. It can be assumed that predominantly nonenhancing thrombi without or with only minor accumulation of the albumin-binding blood pool contrast agent gadofosveset are already older and more organized and thus possibly more stable. This could thus serve as an explanation for the reduced rupture risk associated with nonenhancing thrombi, which we observed in our study. Still, given the ongoing controversy surrounding the effect of intraluminal thrombus on aneurysm stability and rupture, the small sample size and the preclinical setting, it might still be premature to evaluate the prognostic value of nonenhancing thrombus for predicting aneurysm rupture.

The relationship between increased signal enhancement of the albumin-binding probe and AAA progression we observed in our experiments is consistent with the results reported by prior investigators evaluating vascular permeability in an animal model of atherosclerosis^14^. The presence of increased vascular permeability in coronary heart disease was previously established in transmission electron microscopy studies, reporting death of endothelial cells, vascular denudation, and widening of tight junctions^14^.

With its longer intravascular circulation time and higher relaxivity, gadofosveset also allows longer image acquisition times with improved spatial resolution. In this context, Klessen *et al*. reported an improved image quality for 10 ml of gadofosveset compared to 30 ml of gadopentetate diglumine^21^. Clinically, delayed-enhancement MRI with gadofosveset has already been used to characterize vascular wall structures and vulnerable plaques and to quantify and characterize aneurysm sac contents in patients after endovascular repair, providing information on the volumes of unorganized thrombus, organized thrombus, and endoleak^14,22,23^.

A limitation of the present study is that murine and human abdominal aortic aneurysms occur in different anatomic sites (suprarenal versus infrarenal), which may reflect regional differences in collagen and elastin composition and thus give rise to different mechanical properties^24–26^. Compared to other animal models, an advantage of the chosen model is the spontaneous development of aneurysms in response to AngII infusion. Given the insufficiently elucidated mechanism of biological distribution and chemical speciation of gadolinium-based contrast agents^27^ as well as potential nephrotoxity, the use of a gadolinium-based probe might be considered a general drawback of this approach. However, a stable gadopentetate core and phosphodiester linkage to a lipophilic albumin-binding group result in a higher thermodynamic stability and kinetic inertness of the probe used here compared to gadolinium diethylenetriamine penta-acetic acid (DTPA)^28^. While interpreting our data, one must realize, that conclusions are based on small sample sizes as well as a preclinical animal model. Finally, it should be noted that the results of this study may be very specific to the animal model used, whereas there is no clinical evidence for the reported results so far.

While MR-based molecular imaging is still mainly performed with preclinical probes, gadofosveset has the advantage that it is already approved clinically, facilitating the translation of our findings to patients with AAA. Adding to the possibility of clinical translation, gadofosveset was administered at a clinical dose in the present study. Besides, imaging in this study was performed in a clinical 3 Tesla MR scanner. As a result, relaxation, rotational correlation, and signal properties could also be transferred to human application. For these reasons, results from the present study might be easily translated to patients with cardiovascular disease and AAA. Future studies could evaluate the impact this *in vivo* parameter might have on treatment strategies and their outcomes. In the foreseeable future, molecular imaging might therefore assist radiologists and clinicians in the individualized assessment and treatment of AAA.

The present study shows the feasibility of contrast-enhanced MRI using albumin-specific gadofosveset to noninvasively assess endothelial permeability and risk of rupture in murine AAA.

## Materials and Methods

### Study design

Twelve-week-old homozygous male ApoE^−/−^ (B6.129P2-Apo^Etm1Unc^/J) mice were obtained from our Research Institute of Experimental Medicine. Animal care, housing, and all procedures regarding the animals as well as the experimental protocols were performed according to the guidelines and regulations of the Federation of Laboratory Animal Science Associations and the local Guidelines and Provisions for Implementation of the Animal Welfare Act, as approved by the Regional Office for Health and Social Affairs Berlin (LAGeSo).

For aneurysm induction, osmotic minipumps (Alzet, Model-2004, Durect Corporation, USA) were implanted subcutaneously into mice at 12 weeks of age and set to deliver angiotensin II (AngII) at a rate of 1000 ng/kg/min for up to 4 weeks (n = 36)^29^. Sham-treated mice received saline-filled pumps.

In the longitudinal study (study 1), imaging was performed after 1 week before and 30 min after injection of a clinical dose of 0.03 mmol/kg gadofosveset (n = 18). The mice were followed up for 4 weeks with death as endpoint to evaluate the potential of gadofosveset-enhanced MRI to predict aneurysm rupture. In the week-by-week study (study 2), ApoE^−/−^ and sham-treated mice were imaged before and after contrast medium administration, and tissue was harvested at weeks 1, 2, 3 and 4 after AngII infusion (n = 9 per group). Following the imaging session, histology, fluorescence immunohistochemistry, inductively coupled mass spectroscopy (ICP-MS) and laser ablation inductively coupled plasma mass spectrometry (LA-ICP-MS) were performed. The investigators performing these tests were blinded to the intervention (ApoE^−/−^ versus sham-treated ApoE^−/−^ animals), and to aneurysm age (1–4 weeks). Results were reported in accordance with the ARRIVE guidelines.

### *In vivo* magnetic resonance experiments

Imaging was performed in a clinical 3 T Siemens MRI scanner (Biograph-mMR, Siemens Healthcare Solutions, Germany) using a 4-channel receive-coil array for mouse body applications (Mouse Heart Array, P-H04LE-030, Version1, Rapid Biomedical, Germany). After induction of anesthesia, mice were imaged in supine position.

After a three-dimensional (3D) gradient echo scout scan, a non-contrast-enhanced two-dimensional time-of-flight angiography (2D TOF) was acquired in transverse orientation for visualization of the abdominal aorta with the following parameters: field of view (FOV) of 200 × 200 mm, matrix of 960 × 960, resolution of 0.2 × 0.2 × 0.5 mm, 40 slices, repetition time(TR)/echo time (TE) of 35 ms/4.44 ms, flip angle of 90°, and bandwidth of 124 Hz/Px. A maximum intensity projection (MIP) was then automatically generated to obtain an arterial angiogram of the abdominal aorta for planning the subsequent MR angiography and delayed-enhancement scans. A 2D inversion time (TI) scout sequence, planned perpendicular to the abdominal aorta, was used to determine the optimal TI for blood signal nulling. Acquisition parameters were as follows: for the 2D TI sequence - FOV 340 × 340 mm, matrix 576 × 576, spatial resolution 0.6 × 0.6 × 3 mm, TR/TE 44.91 ms/2.09 ms, flip angle 35°, bandwidth 579 Hz/Px, TR between subsequent IR pulses 1000 ms; and for the high-resolution 3D IR FLASH sequence - FOV 57 × 57 mm, matrix 384 × 384, spatial resolution = 0.1 × 0.1 × 0.3 mm, 56 slices, TR/TE = 1019,72/7,02 ms, flip angle 30°, bandwidth 130 Hz/px, TR between subsequent IR pulses = 1000 ms.

To avoid rapid cooling during MRI, the body temperature (37 °C) of the mice was monitored using an MR-compatible heating system (Model 1025, SA Instruments Inc, Stony Brook, NY). For venous access, a small-diameter tube with an attached 30 G cannula was used, enabling the injection of contrast agents into the tail vein.

### Anesthesia and tissue harvesting

Animals were anesthetized by an intraperitoneal injection of a combination of medetomidine (500 μg/kg), midazolam (5 mg/kg), and fentanyl (50 μg/kg). For the implantation of the osmotic minipumps and the longitudinal setup, a reversal agent (atipamezole (2.5 mg/kg), flumazenil (500 μg/kg), and naloxon (1200 μg/kg)) was administered^30^. After anesthesia and euthanasia by cervical dislocation, terminal exsanguination was performed by perfusing the left ventricle with physiological saline for 10 min.

### Evans blue dye

We assessed vascular permeability by quantification of the leakage of Evans Blue Dye into the arterial wall. 0.1 mL of 1% Evans Blue Dye (using phosphate-buffered saline) was injected into the tail vein of ApoE^−/−^ mice (n = 3) and sham-operated ApoE^−/−^ mice (n = 3). After 60 minutes, the AAA segment was isolated, dried, and weighted. The amount of Evans Blue dye was determined by measuring the absorbance of Evans Blue at 620 nm and calculation against the standard curve of known pure Evans blue dye concentrations.

### Immunofluorescence analysis

The abdominal aorta was removed and immediately embedded in tissue-freezing medium (OCT compound) for cryosectioning. Abdominal aortic tissues were cut at −20 °C into 10-µm cryosections and immediately mounted on SuperFrost Plus adhesion slides (Thermo Scientific). Nine individual animals were analyzed for each time point (1, 2, 3, and 4 weeks) and nine individuals each for the sham-treated group. Transverse sections (10 μm thick) were collected from several sites along the abdominal aorta at distances of 200 μm, covering the imaged region. Two representative sections per animal were selected for analysis.

Immunofluorescence staining was performed using polyclonal primary anti-mouse serum antibodies (Goat polyclonal to Mouse Serum Albumin, ab19194, Abcam, Australia, 1:100) incubated with polyclonal secondary antibody labelled with AlexaFluor 568 (goat anti-Rabbit IgG, Thermo Fisher Scientific, Germany, 1:500). Fluorescence-positive regions were assessed on digital images by computerized planimetry with the use of ImageJ (version 1.51, National Institutes of Health). The fluorescence-positive region was segmented and expressed as a percentage of the overall adventitial area. It was matched with LA-ICP-MS images.

### Histological analysis

Cryosections (10 µm) were also stained with hematoxylin and eosin (H&E) for overall visualization of tissue components and with Miller’s EvG for visualization of elastic fibers. Then slices were photographed using a light microscope (Observer Z1, Carl Zeiss Microscopy GmbH, Jena, Germany). Digitized images of H&E, EvG, and immunofluorescence sections were used for vessel lumen and aneurysm size measurements (ImageJ software, Version 1.51).

### Laser ablation inductively coupled plasma mass spectrometry

For elemental bioimaging, LA-ICP-MS was performed using an LSX-213-G2+ laser system (CETAC Technologies, USA), equipped with a two-volume HelExII cell connected via Tygon tubing to an ICP-MS spectrometer (ICPMS-2030, Shimadzu, Japan). Quantification and visualization were performed with an in-house developed software (Robin Schmid, WWU Münster, Germany). Histological samples were ablated via line-by-line scan with a spot size of 15 µm, a scan speed of 45 µm/s, and 800 mL/min helium as carrier gas. Subsequent analysis was conducted in collision gas mode using He as collision gas and 100 ms integration time for the analyzed isotopes ^31^P, ^64^Zn, ^158^Gd, and ^160^Gd. For quantification of Gd, matrix-matched gelatin-based standards were used. Nine gelatin standards (10% w/w), including a blank, were spiked with different Gd concentrations ranging from 1 to 5.000 µg/g. There was good linear correlation for averaged intensities of scanned lines with a regression coefficient R^2^ = 0.9999 over this concentration range. Limit of detection (LOD) and limit of quantification (LOQ), calculated with the 3σ- and 10σ-criteria, were 8 ng/g and 28 ng/g Gd, respectively.

### Inductively coupled plasma mass spectrometry

ICP-MS was used to determine gadolinium concentrations. Half of the abdominal aorta/the AAA was collected and snap-frozen in liquid nitrogen (n = 3 per group). Before ICP-MS, the samples were digested in 300 μL 70% nitric acid at room temperature overnight, followed by dilution in 1 mL of deionized water.

### Magnetic resonance imaging analysis

All MR images were analyzed with Visage (version 7.1, Visage Imaging, San Diego, CA), and all analyses were performed blinded to the intervention (ApoE^−/−^ versus sham-treated animals) and to aneurysm age (1, 2, 3, or 4 weeks). 2D regions of interest (ROIs) were drawn around areas of signal enhancement within the aneurysm borders or the vessel wall in sham-treated animals in delayed-enhancement MR images, anatomically co-localized with high-resolution MR angiography images. In the delayed-enhancement gadofosveset sequences, signal from blood was suppressed.

Contrast-to-noise-ratio (CNR) was calculated using the following formula with noise being defined as the standard deviation in the background ROI placed in air anterior to the aorta.

CNR = (combined vessel wall and aneurysmal aortic tissue signal intensity − blood signal intensity)/standard deviation of image noise.

### Statistical analysis

All statistical analysis was performed with the statistical software “R” (version3.2.2, R Development Core Team, 2015). A priori analysis was performed to determine required group sizes. An effect strength of 0.5 (Cohen’s d) was applied to the main size, with 50% expected/to be detected difference between test and sham-treated group.

Grouped continuous variables were tested for normal distribution with the Shapiro-Wilk test. Continuous variables with normal distribution were compared with Student’s t-test. Continuous variables satisfying the assumptions of normal distribution were examined by one-way analysis of variance (ANOVA). Variables not satisfying the assumption of normality were compared by the Mann-Whitney U-test (two variables). Bar charts with standard deviations were generated for visual illustration of CNR values. To assess the risk of rupture in the longitudinal study, a receiver operating curve (ROC) analysis was performed to determine the optimal ratio of the size of the nonenhancing thrombus area to the CNR for prediction of AAA rupture. Based on this, accuracy and sensitivity and specificity were calculated. Univariate calculations were performed using Pearson’s correlation coefficient.

## Supplementary information

Supplementary Information.

## Data Availability

All data are available in the manuscript and the supplementary material.
