# Author Correction: Noninvasive imaging of vascular permeability to predict the risk of rupture in abdominal aortic aneurysms using an albumin-binding probe

**DOI:** 10.1038/s41598-021-89153-z

**Published:** 2021-04-30

**Authors:** Lisa C. Adams, Julia Brangsch, Carolin Reimann, Jan O. Kaufmann, Kristin Nowak, Rebecca Buchholz, Uwe Karst, Rene M. Botnar, Bernd Hamm, Marcus R. Makowski

**Affiliations:** 1 Charité – Universitätsmedizin Berlin, corporate member of Freie Universität Berlin, Humboldt-Universität Zu Berlin, and Berlin Institute of Health, Charitéplatz 1, 10117 Berlin, Germany; 2 Department of Veterinary Medicine, Institute of Animal Welfare, Animal Behavior and Laboratory Animal Science, Freie Universität Berlin, Königsweg 67, Building 21, 14163 Berlin, Germany; 3 Division 1.5 Protein Analysis, Federal Institute for Materials Research and Testing (BAM), Richard-Willstätter-Str. 11, 12489 Berlin, Germany; 4 Department of Chemistry, Humboldt-Universität zu Berlin, Brook-Taylor-Str. 2, 12489 Berlin, Germany; 5 Institute of Inorganic and Analytical Chemistry, Westfälische Wilhelms-Universität Münster, Corrensstr. 30, 48149 Münster, Germany; 6 King’s College London, School of Biomedical Engineering and Imaging Sciences, United Kingdom, St Thomas’ Hospital Westminster Bridge Road, London, SE1 7EH UK; 7 BHF Centre of Excellence, King’s College London, United Kingdom, Denmark Hill Campus, 125 Coldharbour Lane, London, SE5 9NU UK

Correction to: *Scientific Reports* 10.1038/s41598-020-59842-2, published online 24 February 2020

The original version of this Article omitted affiliations for Jan O. Kaufmann. The correct affiliations for Jan O. Kaufmann are listed below:

Charité –Universitätsmedizin Berlin, corporate member of Freie Universität Berlin, Humboldt-Universität Zu Berlin, and Berlin Institute of Health, Charitéplatz 1, 10117, Berlin, Germany.

Division 1.5 Protein Analysis, Federal Institute for Materials Research and Testing (BAM), Richard-Willstätter-Str. 11, 12489 Berlin, Germany.

Department of Chemistry, Humboldt-Universität zu Berlin, Brook-Taylor-Str. 2, 12489 Berlin, Germany.

This has now been corrected in the HTML and PDF versions of this Article.

