# Supplementary material for: Noninvasive imaging of vascular permeability to predict the risk of rupture in abdominal aortic aneurysms using an albumin-binding probe
Source: Sci Rep. 2020 Feb 24;10:3231. doi: 10.1038/s41598-020-59842-2 (PMC7039902; doi:10.1038/s41598-020-59842-2)
Supplement: Supplementary file 1 — Supplementary Information. [file 41598_2020_59842_MOESM1_ESM.docx]

**Supplementary Information**

**Noninvasive imaging of vascular permeability to predict the risk of rupture in abdominal aortic aneurysms using an albumin-binding probe**

**Authors:**

Lisa C. Adams^1*^, Julia Brangsch^1,2^, Carolin Reimann^1,2^, Jan O. Kaufmann^1^, Kristin Nowak^1^, Rebecca Buchholz^3^, Uwe Karst^3^, Rene M. Botnar^4,5^, Bernd Hamm^1^, Marcus R. Makowski^1,4,5^

**Affiliations:**

^1^Charité – Universitätsmedizin Berlin, corporate member of Freie Universität Berlin, Humboldt-Universität zu Berlin, and Berlin Institute of Health, Charitéplatz 1, 10117 Berlin, Germany

^2^Department of Veterinary Medicine, Institute of Animal Welfare, Animal Behavior and Laboratory Animal Science, Freie Universität Berlin, Königsweg 67, Building 21, 14163 Berlin, Germany

^3^Institute of Inorganic and Analytical Chemistry, Westfälische Wilhelms-Universität Münster, Corrensstr. 30, 48149 Münster, Germany

^4^King’s College London, School of Biomedical Engineering and Imaging Sciences, United Kingdom, St Thomas’ Hospital Westminster Bridge Road, London SE1 7EH, United Kingdom

^5^BHF Centre of Excellence, King’s College London, United Kingdom, Denmark Hill Campus, 125 Coldharbour Lane, London SE5 9NU, United Kingdom


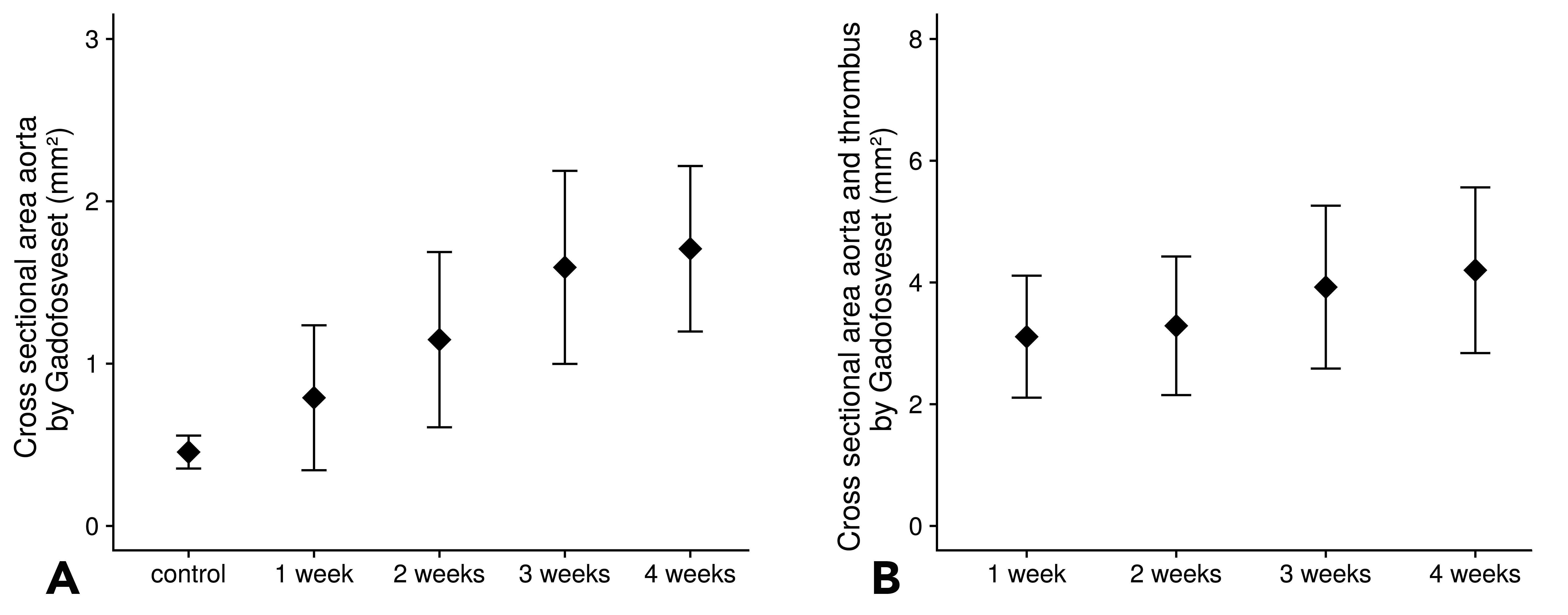


**Figure S1.** In vivo size measurement of abdominal aortic aneurysms and thrombus cross-sectional areas (week-by-week study). (A) In vivo MRI shows an increase in cross-sectional areas representing aortic lumen size over 4 weeks of angiotensin II infusion. (B) In vivo MRI combining cross-sectional areas with associated thrombus areas also show an increase in size over 4 weeks.

**
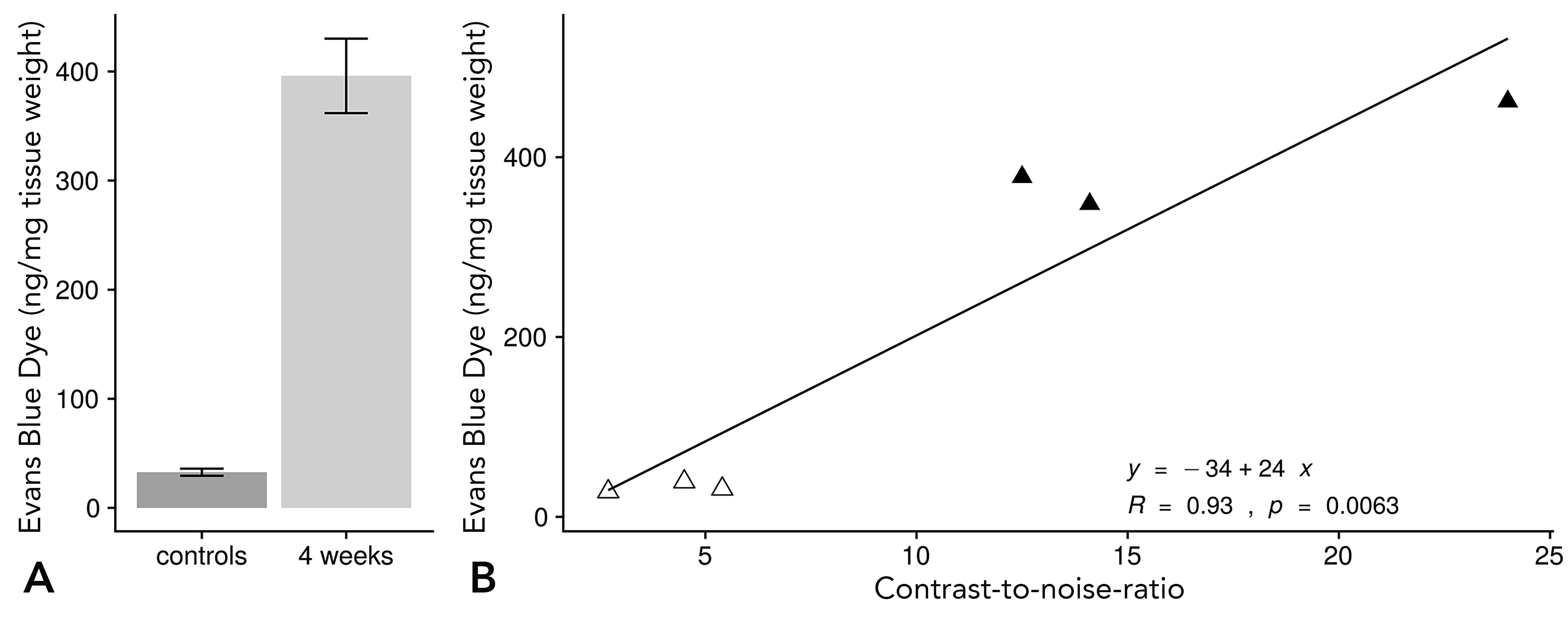
**

**Figure S2.** A) There is a very high uptake of Evans Blue Dye in ApoE^-/-^ mice with 4 weeks of subcutaneous Angiotensin II infusion compared to controls, which only exhibit a minimal uptake of the dye. B) The concentration of Evans Blue Dye in the aortic tissue correlates with in vivo measured contrast-to-noise ratios (n=3 per group).
